# Characterization of genetic diversity on tropical *Trichoderma* germplasm by sequencing of rRNA internal transcribed spacers

**DOI:** 10.1186/s13104-019-4694-1

**Published:** 2019-10-18

**Authors:** Yara Barros Feitosa, Valter Cruz-Magalhães, Ronaldo Costa Argolo-Filho, Jorge Teodoro de Souza, Leandro Lopes Loguercio

**Affiliations:** 1 Center of Nuclear Energy in Agriculture (CENA), State University of São Paulo (USP), Piracicaba, São Paulo 13416-000 Brazil; 2 Department of Biological Sciences (DCB), State University of Santa Cruz (UESC), Ilhéus, Bahia 45662-900 Brazil; 3 Department of Phytopathology (DFP), Federal University of Lavras (UFLA), Lavras, Minas Gerais 37200-000 Brazil

**Keywords:** Biological control, Polymerase chain reaction, Internal transcribed spacer, BlastN, Phylogeny, Biotechnological development

## Abstract

**Objective:**

*Trichoderma* species are found in soil and in association with plants. They can act directly or indirectly in the biological control of plant diseases and in the promotion of plant growth, being among the most used fungi in the formulation of bioproducts applied to agricultural systems. The main objective of this study was to characterize at a first-tier level a collection of 67 *Trichoderma* isolates from various tropical sources, based solely on sequencing of the internal transcribed spacer (ITS) region of the rRNA genes. Our goal was to provide a preliminary idea of the baseline diversity in this collection, to combine this information later with an array of other isolate-specific physiological data. This study provides a required knowledge at molecular level for assessment of this germplasm potential as a source of biotechnological products for beneficial effects in plants.

**Results:**

Sequencing of the ITS region showed that the 67 *Trichoderma* isolates belonged in 11 species: *T. asperellum, T. atroviride, T. brevicompactum, T. harzianum, T. koningiopsis, T. longibrachiatum, T. pleuroticola, T. reesei, T. spirale, T. stromaticum* and *T. virens*. A total of 40.3% of the isolates were very closely related to each other and similar to *T. harzianum*. The baseline genetic diversity found indicates that the collection has different genotypes, which can be exploited further as a source of bioproducts, aiming at providing beneficial effects to plants of interest to cope with biotic and abiotic stresses.

## Introduction

Filamentous fungi of the genus *Trichoderma* (*Ascomycota*) are among the microorganisms most used in biological control of plant diseases [1], displaying mechanisms of action that include direct and indirect antagonism against plant pathogens [2]. *Trichoderma* can establish endophytic associations with plants, systemically induce resistance against phytopathogens, increase nutrient uptake, and consequently promote plant growth [3]. They are also known for their abilities to grow under diverse environmental conditions and to parasitize other fungi [4]. Due to these features, *Trichoderma* can colonize an array of niches and compete with other microorganisms for space, nutrients and light, which explains its success as a biological control agent [5]. All these characteristics, associated with a recognized efficacy in the generation of dispersive propagules (conidia), make *Trichoderma* spp. ideal for the development of bioproducts for beneficial effects on plants [6]. Several abiotic factors (light, temperature, humidity, pH, carbon and nitrogen sources, oxygen) can interact with genetic components (inherent genetic variability of different *Trichoderma* isolates), producing unique growth and sporulation responses, which tend to contribute to the biocontrol efficacy in a strain-specific manner [7–9]. The internal transcribed spacer (ITS) region of rRNA genes is considered the primary barcode sequence for evolutionary/phylogenetic studies on fungi, due to its relevant features such as (i) ease of amplification in a very reproducible manner (ii) a widespread use among a variety of fungal systems (iii) a tendency of displaying a large barcode gap, (iv) the possibility of alignment across all the kingdom, and (v) an appropriate length for amplification, sequencing and phylogenetic information supply [10, 11]. In this study, direct amplicon sequencing of the ITS region of rDNA was used to preliminarily characterize a tropical collection of 67 *Trichoderma* isolates from the Atlantic and Amazon rainforests, and ‘caatinga’ (semi-arid) biomes of Brazil. This collection has been evaluated concerning isolate-specific phenotypes related to growth and sporulation levels responsive to biotic and abiotic factors [12]. Based on the molecular characterization of *Trichoderma* isolates described in the present work, further studies on this germplasm will address its potential as a source of new bioproducts to be used in biological control of plant diseases and other beneficial effects.

## Main text

### Methods

#### Trichoderma isolates and growth conditions

The *Trichoderma* collection studied consisted of 67 isolates obtained from different natural environments (biomes) in different provinces of Brazil, including Bahia (47), Amazonas (9), Rondônia (10) and Minas Gerais (1) (Additional file 1: Table S1). The isolates were cultured in Petri dishes containing PDA medium (Difco™) and incubated at 25 °C under constant light. After 5 days, the conidia were collected and stored in sterile glycerol (50%) at − 80 °C.

#### Genomic DNA extraction and amplification

For a first-tier molecular identification of *Trichoderma* species and an assessment of the baseline diversity of isolates within the collection, the ITS region of the rDNA was sequenced. Each isolate was grown in 30 mL of PD medium (inside 50-mL Falcon tubes) at 25 °C under constant light for 4 days in a growth chamber. For genomic DNA extraction, an initial step of physical mycelium break was employed through addition of 7–10 mL of sterile glass beads in each tube and vortexing for 2 min. Afterwards, 13 mL of each grinded mycelium suspension was transferred to clean 50-mL tubes and centrifuged for 5 min at 13,400 rpm. A wet weight of 200 mg for each pellet was then used for total DNA extraction with the DNeasy^®^ Plant Mini Kit (Qiagen™) following the manufacturer’s protocol. The ITS region was amplified by PCR using the ITS-1 [13] and ITS-4 [14] universal primers. The PCR mixture (40 μL) contained 0.5 μL of 2 U µL^−1^ Taq DNA polymerase (Invitrogen™), 1.2 μL of 1.5 mM MgCl_2_, 1.67 μL of each forward and reverse primers (both as 20-μM solutions), 1 μL of 1 mM dNTPs (250 µM each), 4 μL of 10 × buffer, 3.4 μL of DNA template (~ 10–30 ng) and 26.56 μL of distilled water. Amplifications were performed in 0.2-mL PCR tubes using an Applied Biosystems Veriti Thermal Cycler. The conditions for PCR amplification were 95 °C for 5 min, followed by 35 cycles of 95 °C for 30 s, 55 °C for 30 s and 72 °C for 30 s, and a final extension at 72 °C for 10 min. PCR products were visualized on 1.5% agarose gel and the amplified DNA was purified from the gel using the PureLink^®^ Quick Gel Extraction Kit (Invitrogen™) following the manufacturer’s instructions.

#### Internal transcribed spacer (ITS) sequencing

Direct sequencing of the purified amplicons was performed by the Sanger method using the ABI-PRISM^®^ 3100 Genetic Analyzer system. Sequencing reactions in final volumes of 10 μL contained 3 μL of BigDye Terminator v3.1 Cycle Sequencing RR-100, 5 ng μL^−1^ of DNA template, and 0.25 pmol μL^−1^ of each ITS1 or ITS4 primers. Sequencing amplifications were performed in a GeneAmp PCR System 9700 thermocycler as follows: 3 min at 96 °C, plus 25 cycles of 10 s at 96 °C, 5 s at 55 °C and 4 min at 60 °C. The reaction products were precipitated with 40 μL 75% isopropanol (3:1, v/v) for 20-30 min at room temperature, centrifuged at 13,000 rpm for 15 min at 4 °C, washed with 200 μL 60% ethanol (quick vortexing), and centrifuged at 13,000 rpm for 5 min at 4 °C. The pellets were air-dried, resuspended in 10 μL of Hi-Di formamide, denatured at 95 °C for 5 min, cooled on ice for 5 min and electro-injected in the automatic sequencer. The sequencing data were collected using the Data Collection v 1.0.1 program. At least two sequencing reactions were performed for each amplicon/isolate.

#### Phylogenetic and diversity analyses

Sequences from the ITS region of the isolates were compared to those in GenBank (NCBI) through the BlastN program [15]. From these results, reference sequences for the corresponding *Trichoderma* species (Additional file 2: Table S2) were used to construct multiple sequence alignments (MSA). For the phylogenetic approach, the GUIDANCE2 server [16] with the MAFFT alignment algorithm [17] was used to ensure that only positions with high probabilities of being correctly aligned were used. Phylogenetic analysis was performed with the MEGA7 program [18] using the Maximum Likelihood (ML) method. The analysis involved a total of 80 DNA sequences, 67 for our study and 13 type species from the database. The tree was edited using the FigTree program [19].

### Results

**Fig. 1:**
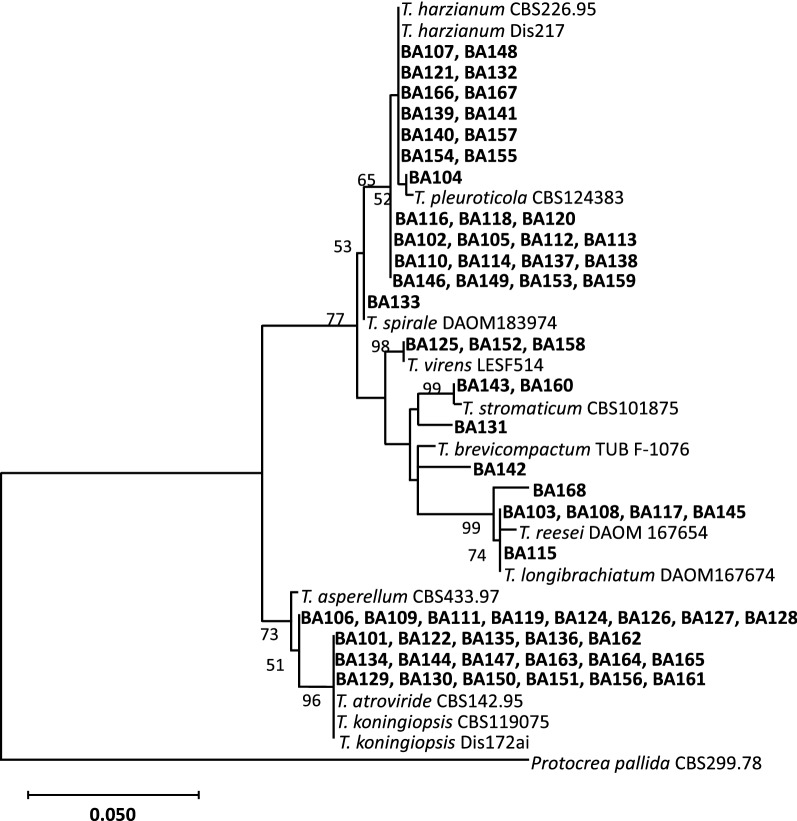
Phylogenetic tree of *Trichoderma* isolates from tropical sources based on sequences of the ITS region. The tree was constructed by using the Maximum Likelihood method with 425 aligned nucleotides from the ITS region of the rDNA. The nucleotide substitution model used was GTR + G to model evolutionary rate differences among sites [18]. The bootstrap analysis was performed with a 1000 resamplings and values above 50% are shown on the appropriate branches. The ITS sequence from *Protocrea pallida* was used as the outgroup. The list of (i) ITS sequences with accession numbers of the ‘BA’ isolates (Additional file 1: Table S1) and (ii) type species/ITS sequences with accession numbers used in the alignments (Additional file 2: Table S2) are shown in additional material. The scale bar represents the number of expected substitutions per site

In order to assess the baseline diversity within the *Trichoderma* collection of this study, a phylogeny-based analysis on the ITS region was performed. The results showed that the 67 isolates from a tropical origin clustered with the reference sequences in variable manners (Fig. 1). The clusters ranged from a pairwise configuration between an isolate and a reference sequence to groups with many isolates and more than one reference sequence. The isolates from the collection were more closely related to 11 species of the *Trichoderma* genus: *T. asperellum, T. atroviride, T. brevicompactum, T. harzianum, T. koningiopsis, T. longibrachiatum, T. pleuroticola, T. reesei, T. spirale, T. stromaticum* and *T. virens*. In this context, the distribution of isolates among the species was heterogeneous. The largest group comprising 27 isolates (40.3%) was related to the *T. harzianum* species complex; 12 of them (17.9%; BA107, BA121, BA166, BA139, BA140, BA154, BA148, BA132, BA167, BA141, BA157, BA155) grouped within the cluster containing the two *T. harzianum* references (‘CBS226.95’ and ‘Dis217’), and 15 (22.4%; BA116, BA102, BA110, BA146, BA118, BA105, BA114, BA149, BA120, BA112, BA137, BA153, BA113, BA138, BA159) were more related to this cluster (Fig. 1); due to the not so close relationship to the *T. harzianum* reference strains, this latter group of 15 isolates were indicated as ‘*Trichoderma* sp-1’ (Additional file 1: Table S1; also see below). In terms of a first-tier, preliminary identification of the isolates based on the ITS sequences, some noteworthy certainties were verified (Fig. 1; Additional file 1: Table S1): (i) the isolate BA104 was most closely related to the *T. pleuroticola* species (branching out from the *T. harzianum*’s complex); (ii) BA133 belonged in the *T. spirale* species; (iii) BA125, BA152 and BA158, grouped with *T. virens*; (iv) BA143 and BA160 were most closely related to *T. stromaticum*; and (v) BA115 belonged in the *T. longibrachiatum*.

The other isolates, on their turn, showed a pattern of uncertain relationship with the reference isolates, with variable levels of proximity. For instance, a cluster with 17 isolates (25.4%) could not be distinguished from three reference sequences, the ‘CBS142.95’ of *T. atroviride* and the ‘CBS119075’ and ‘Dis172ai’ of *T. koningiopsis* (Fig. 1; bottom part of the dendrogram). BA131, BA142 and BA168, and a group of eight isolates (including BA106 to BA128; Fig. 1) did not form a cluster with any reference sequence. For an intra-collection differentiation, those isolates with uncertain species assignment were given an ‘sp’ indication, followed by specific extension numbers to represent distinct locations in the dendrogram topolgy (Additional file 1: Table S1). The *T. brevicompactum, T. asperellum* and *T. reseei* reference sequences (‘TUB F-1076’, ‘CBS433.97’, ‘DAOM 167654’, respectively) did not specifically clustered with any isolate, although the second one appeared more closely related to BA142, and the last one to BA103, BA108, BA117 and BA145 isolates (Fig. 1).

### Discussion

The development of *Trichoderma*-based bioproducts begins with screening procedures over a hopefully variable genetic material available from collection-assembly efforts [6, 20], as the identification of novel genotypic variations among isolates allows to increase the options for beneficial-effects applications [21–23]. The tropical collection of *Trichoderma* here studied bears some genetic diversity on an isolate-specific level, based on a phylogenetic assessment with ITS sequences (Fig. 1). Despite that ITS is the primary barcode sequence for fungi, bearing several relevant features for phylogenetic/evolutionary studies in fungi [10, 11], alone it is not a sufficient marker for full species discrimination in highly speciose genera, such as *Trichoderma* [24]. Therefore, not unexpectedly, the level of similarities among all the ITS sequences was high, with differences within a range of 0.05 to 0.10 substitutions per site. However, the clustering pattern provided by the dendrogram topology suggest that the tropical isolates of this collection may be possibly assigned to more than the 11 species indicated by the reference strains (Fig. 1). The fact that more than a third of the isolates were genetically related to *T. harzianum* may be explained by the recognized condition of this taxon as a complex of cryptic species [25], in which the analysis based on ITS sequences is not able to distinguish closely related species in this complex [26]. The 11 species of *Trichoderma* identified/characterized in this study, coupled with the isolate-specific phenotypic aspects related to growth and sporulation responses to biotic and abiotic factors [12], suggest that the genetic diversity associated with this germplasm (at the genotypic level) will be useful for further work aiming at developing novel bioproducts with an array of possible applications. Subsequent characterization steps based on assays at laboratory and agronomic scales are needed to evaluate which isolates and conditions stand out, such as for biocontrol of phytopathogens or plant-growth promotion for distinct plant species. Once particular strains of this collection are revealed as promising sources for beneficial-effects related bioproducts, fine-tuned species definition can be achieved by characterizing other genes [24], as an aid to the ITS sequence database here provided.

## Limitations

The main limitation of this study is the fact that the information contained in the ITS region is not sufficient to separate some of the isolates at the species level within the genus *Trichoderma*. However, since full, unequivocal taxonomy was not the main goal of this study, we claim that ITS-based preliminary characterization of *Trichoderma* isolates, coupled with phenotypic differences detected through specific comparative assays (e.g. [12, 21, 23]), was sufficiently appropriate to provide information on the existing diversity in this collection at the isolate (genotypic) level.

## Supplementary information

**Additional file 1: Table S1.** Internal Transcribed Spacer (ITS) sequences from the 67 tropical *Trichoderma* isolates from the ‘BA’ series and the corresponding *GenBank* accession numbers.

**Additional file 2: Table S2.** List of ITS sequences from type species used in this study.

## Data Availability

The data sets and working sheets are available upon request to Dr. Valter Cruz-Magalhães (E-mail: valter.magalhães@ufla.br) and Dr. Leandro L. Loguercio (E-mail: leandro@uesc.br).

## Abbreviations

ITS: internal transcribed spacer
PDA: potato dextrose agar
PCR: polymerase chain reaction
dNTPs: deoxynucleosides triphosphate
MSA: multiple sequence alignment
NCBI: National Center for Biotechnology Information
