# Supplementary material for: Characterization of genetic diversity on tropical Trichoderma germplasm by sequencing of rRNA internal transcribed spacers
Source: BMC Res Notes. 2019 Oct 18;12:663. doi: 10.1186/s13104-019-4694-1 (PMC6798453; doi:10.1186/s13104-019-4694-1)
Supplement: Supplementary file 1 — Additional file 1: Table S1. Internal Transcribed Spacer (ITS) sequences from the 67 tropical Trichoderma isolates from the ‘BA’ series and the corresponding GenBank accession numbers. [file 13104_2019_4694_MOESM1_ESM.docx]

**Additional file 1: Table S1.** Internal Transcribed Spacer (ITS) sequences from the 67 tropical *Trichoderma* isolates from the ‘BA’ series and the corresponding *GenBank* accession numbers the ‘BA’ isolates.

| **Isolates** | **Accession no.** | **Probable species** *^1^* | **Biome** |
| --- | --- | --- | --- |
| BA101 | MK613142 | *Trichoderma* sp-5. | Atlantic forest |
| BA102 | MK613143 | *Trichoderma* sp-1. | Atlantic forest |
| BA103 | MK613144 | *T. reesei* | Atlantic forest |
| BA104 | MK613145 | *T. pleuroticola* | Atlantic forest |
| BA105 | MK613146 | *Trichoderma* sp-1. | Atlantic forest |
| BA106 | MK613147 | *Trichoderma* sp-4. | Atlantic forest |
| BA107 | MK613148 | *T. harzianum* | Atlantic forest |
| BA108 | MK613149 | *T. reesei* | collection (*ex-situ*) *^2^* |
| BA109 | MK613150 | *Trichoderma* sp-4. | Atlantic forest |
| BA110 | MK613151 | *Trichoderma* sp-1. | Atlantic forest |
| BA111 | MK613152 | *Trichoderma* sp-4. | Atlantic forest |
| BA112 | MK613153 | *Trichoderma* sp-1. | Atlantic forest |
| BA113 | MK613154 | *Trichoderma* sp-1. | Atlantic forest |
| BA114 | MK613155 | *Trichoderma* sp-1. | Atlantic forest |
| BA115 | MK613156 | *T. longibrachiatum* | Atlantic forest |
| BA116 | MK613157 | *Trichoderma* sp-1. | Atlantic forest |
| BA117 | MK613158 | *T. reesei* | Atlantic forest |
| BA118 | MK613159 | *Trichoderma* sp-1. | Atlantic forest |
| BA119 | MK613160 | *Trichoderma* sp-4. | Atlantic forest |
| BA120 | MK613161 | *Trichoderma* sp-1. | Atlantic forest |
| BA121 | MK613162 | *T. harzianum* | Atlantic forest |
| BA122 | MK613163 | *Trichoderma* sp-5. | Atlantic forest |
| BA124 | MK613164 | *Trichoderma* sp-4. | Atlantic forest |
| BA125 | MK613165 | *T. virens* | Atlantic forest |
| BA126 | MK613166 | *Trichoderma* sp-4. | Atlantic forest |
| BA127 | MK613167 | *Trichoderma* sp-4. | Atlantic forest |
| BA128 | MK613168 | *Trichoderma* sp-4. | Atlantic forest |
| BA129 | MK613169 | *Trichoderma* sp-5. | Atlantic forest |
| BA130 | MK613170 | *Trichoderma* sp-5. | Atlantic forest |
| BA131 | MK613171 | *Trichoderma* sp-2. | Caatinga |
| BA132 | MK613172 | *T. harzianum* | Caatinga |
| BA133 | MK613173 | *T. spirale* | Caatinga |
| BA134 | MK613174 | *Trichoderma* sp-5. | Amazon rainforest |
| BA135 | MK613175 | *Trichoderma* sp-5. | Amazon rainforest |
| BA136 | MK613176 | *Trichoderma* sp-5. | Amazon rainforest |
| BA137 | MK613177 | *Trichoderma* sp-1. | Amazon rainforest |
| BA138 | MK613178 | *Trichoderma* sp-1. | Amazon rainforest |
| BA139 | MK613179 | *T. harzianum* | Amazon rainforest |
| BA140 | MK613180 | *T. harzianum* | Amazon rainforest |
| BA141 | MK613181 | *T. harzianum* | Amazon rainforest |
| BA142 | MK613182 | *T. brevicompactum* | Amazon rainforest |
| BA143 | MK613183 | *T. stromaticum* | Amazon rainforest |
| BA144 | MK613184 | *Trichoderma* sp-5. | Atlantic forest |
| BA145 | MK613185 | *T. reesei* | Atlantic forest |
| BA146 | MK613186 | *Trichoderma* sp-1. | Atlantic forest |
| BA147 | MK613187 | *Trichoderma* sp-5. | Atlantic forest |
| BA148 | MK613188 | *T. harzianum* | Amazon rainforest |
| BA149 | MK613189 | *Trichoderma* sp-1. | Amazon rainforest |
| BA150 | MK613190 | *Trichoderma* sp-5. | Amazon rainforest |
| BA151 | MK613191 | *Trichoderma* sp-5. | Amazon rainforest |
| BA152 | MK613192 | *T. virens* | Amazon rainforest |
| BA153 | MK613193 | *Trichoderma* sp-1. | Amazon rainforest |
| BA154 | MK613194 | *T. harzianum* | Amazon rainforest |
| BA155 | MK613195 | *T. harzianum* | Amazon rainforest |
| BA156 | MK613196 | *Trichoderma* sp-5. | Amazon rainforest |
| BA157 | MK613197 | *T. harzianum* | Atlantic forest |
| BA158 | MK613198 | *T. virens* | Atlantic forest |
| BA159 | MK613199 | *Trichoderma* sp-1. | Atlantic forest |
| BA160 | MK613200 | *T. stromaticum* | Atlantic forest |
| BA161 | MK613201 | *Trichoderma* sp-5. | Caatinga |
| BA162 | MK613202 | *Trichoderma* sp-5. | Caatinga |
| BA163 | MK613203 | *Trichoderma* sp-5. | Caatinga |
| BA164 | MK613204 | *Trichoderma* sp-5. | Caatinga |
| BA165 | MK613205 | *Trichoderma* sp-5. | Caatinga |
| BA166 | MK613206 | *T. harzianum* | Caatinga |
| BA167 | MK613207 | *T. harzianum* | Caatinga |
| BA168 | MK613208 | *Trichoderma* sp-3. | Caatinga |

*1* In order to provide an intra-collection differentiation for those isolates which did not cluster with any reference strain in Figure 1, a numbered extension to the ‘sp’ indication was given. The different ‘sp’ extension numbers correlate with distinct positions and clusters found for the isolates in the dendrogram, and indicate the corresponding isolates do belong in different, though unknown species. On the other hand, due to the inherent uncertainties given by the dendrogram topology, ‘sp’ isolates with the same extension number does not necessarily belong in the same species. Based on the topology/clustering pattern observed, the following list correspond to the nearest species identified to an ‘sp’ isolate (see Fig. 1): ‘sp-**1**’: *T. harzianum*; ‘sp-**2**’: *T. stromaticum*; ‘sp-**3**’: *T. reesei / longibrachiatum*; ‘sp-**4**’: *T. asperellum*; ‘sp-**5**’: *T. atroviride / koningiopsis*.

*2* The *ex-situ* collection from which the BA108 was obtained is located in the Federal University of Viçosa, Minas Gerais state, Brazil.
