# Supplementary material for: Characterization of genetic diversity on tropical Trichoderma germplasm by sequencing of rRNA internal transcribed spacers
Source: BMC Res Notes. 2019 Oct 18;12:663. doi: 10.1186/s13104-019-4694-1 (PMC6798453; doi:10.1186/s13104-019-4694-1)
Supplement: Supplementary file 2 — Additional file 2: Table S2. List of ITS sequences from type species used in this study. [file 13104_2019_4694_MOESM2_ESM.docx]

**Additional file 2.:**

**Table S2.** List of ITS sequences from type species used in this study

| **Species** | **Isolate** | **Reference** | **Accession no.** |
| --- | --- | --- | --- |
| *T. asperellum* | CBS 433.97^T^ | Holmes et al., 2004 | NR_130668 |
| *T. atroviride* | CBS 142.95^T^ | Holmes et al., 2004 | AY380906 |
| *T. brevicompactum* | TUB F-1076^T^ | Druzhinina et al., 2005 | NR_138434.1 |
| *T. harzianum* | CBS 226.95^T*^ | Chaverri et al., 2015 | AY605713 |
| *T. harzianum* | DIS 217A | Chaverri et al., 2015 | FJ442243 |
| *T. koningiopsis* | GJS 93-20^T^ | Samuels et al., 2006 | NR_131281 |
| *T. koningiopsis* | DIS 172AI | Samuels et al., 2006 | DQ313138 |
| *T. longibrachiatum* | DAOM167674 | Hoyos-Carvajal et al., 2009 | EU280099 |
| *T. pleuroticola* | CBS 124383^T^ | Chaverri et al., 2011 | NR_134420 |
| *T. reesei* | DAOM167654^T^ | Kuhls et al., 1996 | NR_120297 |
| *T. spirale* | DAOM183974^T^ | Hoyos-Carvajal et al., 2009 | NR_077177 |
| *T. stromaticum* | GJS 97-183^T^ | Samuels et al., 2000 | NR_077128 |
| *T. virens* | LESF514 | Montoya et al., 2016 | KT278885 |
| *Protocrea pallida* | CBS 299.78 | Jaklitsch et al., 2008 | NR_111329.1 |

^T^ indicates the type strain of the species; ^T*^ indicates the ex-neotype strain.

CBS = Fungal Biodiversity Centre Culture Collection, the Netherlands; DAOM = Agriculture and Agri-Food Canada National Mycological Culture Collection; G.J.S.= G.J. Samuels; DIS= H.C. Evans endophyte cultures; LESF = Laboratory of Ecology and Systematics of Fungi, Rio Claro, Brazil; TUB = Technical University of Budapest Culture Collection, Laboratory of Industrial Microbiology Szent, Budapest, Hungary.
